# Hexagonal Boron Nitride Nanosheets: Properties, Preparation and Applications in Thermal Management

**DOI:** 10.3390/nano16020101

**Published:** 2026-01-12

**Authors:** Min Liu, Yilin Wang

**Affiliations:** 1 School of Integrated Circuits, Shandong University, Jinan 250100, China; yilinwang@sdu.edu.cn; 2 Shenzhen Research Institute of Shandong University, Shenzhen 518057, China

**Keywords:** boron nitride nanosheets, BNNSs/polymer composite, thermal management, thermal interface materials

## Abstract

Hexagonal boron nitride nanosheets (BNNSs) have emerged as one of the most promising materials for next-generation thermal management, driven by the intensifying heat dissipation demands of highly integrated electronics. While conventional polymer-based packaging materials are lightweight and electrically insulating, their intrinsically low thermal conductivity severely limits effectiveness in high-power devices. The remarkable thermal transport, wide bandgap, chemical robustness, and mechanical strength of BNNSs offer a compelling solution. This review provides a comprehensive overview of the structural and physical foundations that underpin the anisotropic yet exceptional thermal properties of bulk h-BN and BNNSs. We examine major synthesis routes including tape exfoliation, ball milling, liquid-phase exfoliation, chemical vapor deposition, and metal–organic chemical vapor deposition, highlighting how process mechanisms govern nanosheet thickness, defect density, crystallinity, and scalability. Particular emphasis is placed on the advantages of BNNSs in thermal management systems, from their use as high-efficiency thermally conductive fillers and advanced thermal interface materials. We conclude by examining key challenges including large-area growth, filler alignment, and interfacial engineering, and by presenting future research directions that could enable the practical deployment of BNNSs-based thermal management technologies.

## 1. Introduction

With the continuous scaling of integrated circuits (ICs), increasingly more transistors are integrated per unit area. As electrical current flows through resistive components, heat is inevitably generated, leading to significant heat accumulation in highly integrated devices and resulting in extremely high local power densities [1]. Such rapid heat buildup not only degrades the performance of electronic components but also poses severe reliability challenges. Elevated operating temperatures are well known to accelerate device degradation and shorten operational lifetimes. Therefore, effective thermal management has become essential for modern ICs, and the development of next-generation thermal management materials with excellent comprehensive properties is critical for ensuring efficient heat dissipation.

Polymer-based packaging materials, such as epoxy resins, are widely used in IC packaging due to their low density, chemical resistance, electrical insulation, good processability, and low cost. These materials simultaneously serve as protective encapsulants and as thermal management components. However, most polymers possess intrinsically low thermal conductivity (0.1–0.5 W/m·K), which significantly limits their effectiveness in high-power electronic packaging [2]. Introducing inorganic fillers, such as silicon oxide (SiO_2_), silicon carbide (SiC), aluminum oxide (Al_2_O_3_), etc., into polymer matrices is a common strategy to enhance thermal conductivity, yet even at high loadings these composites typically cannot exceed thermal conductivities above 10 W/m·K due to the modest intrinsic conductivity of such fillers.

Since the discovery of graphene in 2004, two-dimensional (2D) materials have attracted significant interest owing to their exceptional electrical, thermal, optical, and mechanical properties, and have shown great potential applications in various fields. Graphene possesses an ultrahigh thermal conductivity of 2000–5300 W/m·K, which is one of the highest thermally conductive materials, and thus graphene is highly attractive for thermal interface materials (TIMs) and heat spreaders [3]. Hexagonal boron nitride (h-BN), which has a similar structure as graphene and is also referred to as “white graphene”, exhibits a theoretically predicted thermal conductivity of 2000 W/m·K [4]. Experimentally, monolayer BN nanosheets (BNNSs) have demonstrated thermal conductivity as high as 750 W/m·K [5], which is nearly twice that of bulk h-BN (390 W/m·K) [6]. Moreover, in contrast to graphene, h-BN is an electrical insulator and is highly stable in air. Further, BNNSs incorporated into polymers can significantly enhance composite thermal conductivity while maintaining electrical insulation. These desirable characteristics position h-BN as one of the most promising candidates for next-generation thermal management materials [7,8,9,10,11,12].

In this review, we first summarize the structural characteristics and fundamental physical properties of bulk h-BN and BNNSs. We then examine recent advances in the synthesis of BNNSs through both top-down and bottom-up approaches, highlighting their growth mechanisms, advantages, and limitations. Subsequently, we focus on the applications of BNNSs in thermal management, including their roles as thermally conductive fillers and thermal interface materials. Finally, we provide a perspective on current challenges and future research directions for the integration of BNNSs into practical thermal management technologies.

## 2. Structure and Physical Properties

### 2.1. Structure

**Figure 1:**
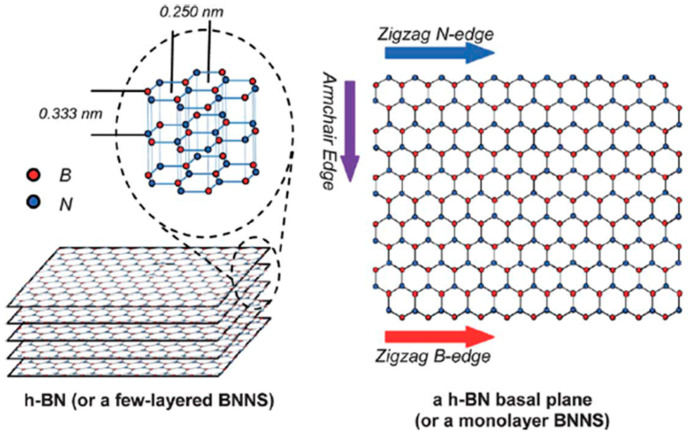
Structure of hexagonal boron nitride (h-BN) [6].

Boron nitride (BN) exhibits in mainly four allotropes: hexagonal BN (h-BN), cubic BN (c-BN), rhombohedral BN (r-BN), and wurtzite BN (w-BN), arising from different bonding configurations associated with *sp*^2^- or *sp*^3^-hybridized B-N orbitals [13]. Among these, h-BN is the most thermodynamically stable phase under ambient conditions. As illustrated in Figure 1, h-BN adopts a layered structure analogous to graphene, with alternating boron and nitrogen atoms arranged in a honeycomb lattice. The in-plane lattice constant of h-BN is 2.50 Å (compared with 2.46 Å for graphene). Adjacent layers are bonded via van der Waals (vdW) interactions [14].

Unlike the purely covalent C-C bonds in graphene, B-N bonds in h-BN possess partial ionic character due to the electronegativity difference between boron and nitrogen. Consequently, multilayer h-BN exhibits AA’ stacking, where each boron atom is stacked directly on top of each nitrogen atom in successive layers and vice versa for nitrogen atom. The lip–lip interaction between adjacent layers causes a stronger interlayer interaction, thereby resulting in slightly smaller interlayer distance (3.33 Å) of h-BN than that of graphite (3.34 Å) [6].

The BNNSs, atomically thin layers of h-BN, retain the same structure as bulk h-BN. Their edges can terminate in either zigzag or armchair configurations [6]. The zigzag edge terminates in a sawtooth pattern of either boron or nitrogen atoms, giving rise to inherently polar boundaries. The armchair edge terminates in a smoother, symmetric row of B-N dimers. These seemingly subtle structural differences in the two edge configurations are sufficient to tail the electronic, optical, chemical reactivity properties of BNNSs.

### 2.2. Physical Properties

In contrast to graphene, h-BN is an electrical insulator with a wide bandgap of ~6 eV [15] and resistivity exceeding 10^16^ Ω cm. Monolayer h-BN exhibits a direct bandgap, whereas bulk h-BN has an indirect gap, the bandgap magnitude is largely insensitive to thickness [16]. Such wide bandgap corresponds to an emission around 215–220 nm, making h-BN a promising material for deep-ultraviolet (DUV) optoelectronics. In addition, h-BN features a low dielectric constant (~3.9) and high dielectric breakdown strength (~8 MV/cm), making it an excellent gate dielectric or tunneling barrier material [17].

Moreover, h-BN is also well known for its exceptional thermal and chemical stability. Thermally, h-BN maintains structural integrity and chemical inertness in air up to ~1000 °C, in vacuum up to ~1400 °C, and in inert atmospheres up to ~2800 °C. The h-BN melts only above ~3000 °C under nitrogen-rich conditions [14]. Chemically, h-BN is resistant to most acids, bases, and organic solvents, does not oxidize up to ~850–900 °C in air, and shows negligible reactivity with molten metals. Further, h-BN exhibits excellent thermal conductivity. The in-plane thermal conductivity of highly oriented pyrolytic h-BN reaches 390 W/m·K [18] at room temperature, comparable to copper, but with significantly lower density (2.1 g/cm^3^). Isotopic purity has a profound influence on the intrinsic thermal conductivity of h-BN. Natural h-BN materials are made with two stable B isotopes (^10^B and ^11^B in an approximate 20:80 ratio), monoisotopic boron (^10^B) significantly enhances in-plane thermal conductivity of h-^10^BN to 585 W/m·K due to the significant reduction in phonon scattering [19]. However, the through-plane thermal conductivity of h-BN is markedly lower, typically on the order of 2–10 W/m K [19,20]. Such strong anisotropic thermal transport originates from the layered crystal structure and directional bonding characteristics: strong in-plane B-N covalent bonds support efficient phonon propagation within the basal plane, whereas phonon transport perpendicular to the layers is severely limited by weak vdW interactions between adjacent sheets.

Compared with bulk h-BN, BNNSs not only retain the intrinsic merits of the parent material, such as wide bandgap, excellent thermal stability and strong chemical inertness, but also exhibit several enhanced properties that emerge in the atomically thin limit. Monolayer h-BN shows an exceptionally high thermal conductivity of ~750 W/m·K at room temperature [5], which can be further enhanced to ~1000 W/m·K at room temperature for isotopically pure h-^11^BN [21]. As the layer thickness increases, the thermal conductivity of h-BN gradually decreases (e.g., ~650 W/m·K for bilayer and ~600 W/m·K for trilayer) [5,22], primarily due to the onset of interlayer phonon scattering. These values still significantly exceed those of bulk h-BN.

In addition to thermal transport advantages, BNNSs demonstrate exceptional mechanical properties. Experimental studies report an ultrahigh Young’s modulus of ~0.8–1 TPa, an intrinsic tensile strength of ~100–130 GPa, and a high fracture strain of ~20–25% [14,23], indicating that BNNSs possess both extreme stiffness and notable flexibility. The unique combination of high thermal conductivity, mechanical robustness, excellent electrical insulation and low density makes BNNSs outstanding candidates for lightweight reinforcement in polymer composites, protective coatings, and other advanced structural and thermal management applications.

## 3. Preparation of BNNSs

The large-scale synthesis of high-quality BNNSs is essential for their practical application in thermal management. Current fabrication approaches can be broadly classified into top-down exfoliation and bottom-up synthesis. Each approach offers distinct advantages and limitations in terms of lateral size, thickness control, defect density, production yield and scalability, all of which directly influence the thermal and mechanical performance of BNNSs-based materials [7,9,13,14,24,25,26,27,28]. Table 1 compares the advantages, disadvantages, and applications of various methods for obtaining BNNSs. In this section, we summarize the most commonly reported preparation methods of BNNSs from the literature to provide background for subsequent discussion.

### 3.1. Top-Down Approaches

Because the interlayer interactions in h-BN consist of vdW forces with partial ionic character, its layered structure can be exfoliated in a manner analogous to graphene. The exfoliation energy of monolayer h-BN is approximately 28 meVÅ^−2^ [29], which is larger than that of graphene, making efficient BNNSs production more challenging and motivating the development of improved exfoliation strategies. According to the exfoliation methods, the top-down approaches can be divided into tape exfoliation, ball milling, liquid-phase exfoliation [13,14,30].

#### 3.1.1. Tape Exfoliation

**Figure 2:**
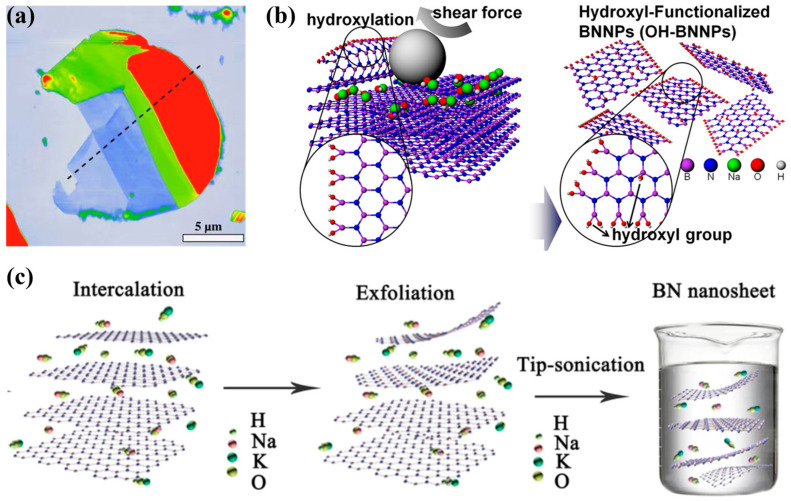
(**a**) AFM topography image of tape-exfoliated BNNSs [32]. (**b**) Schematic diagram of the ball milling process for peeling off and functionalizing the BNNSs [35]. (**c**) Schematic diagram of the intercalation-assisted exfoliation process [44].

Tape exfoliation, originally developed for isolating monolayer graphene [31], has been broadly applied to the preparation of other 2D materials, including BNNSs [32]. In this method, adhesive tape, commonly known as Scotch tape, is repeatedly pressed against bulk h-BN crystals to peel off thin flakes, which are subsequently transferred to a target substrate, as shown in Figure 2a.

The tape exfoliation method is simple and low-cost laboratory implementation without specialized equipment, and produces structurally pristine, atomically smooth, and nearly defect-free nanosheets with lateral dimensions of tens of micrometers, which represent the highest-quality samples available. The exfoliated high-quality BNNSs preserve the intrinsic properties of h-BN, and are ideal for fundamental studies and device prototyping. However, the method suffers from extremely low yield, typically only a few flakes per exfoliation cycle, making it unsuitable for scalable production.

#### 3.1.2. Ball Milling

Ball milling is a versatile mechanical exfoliation technique in which repeated high-energy collisions between grinding media and powders generate shear and compressive forces that overcome interlayer vdW interactions, producing BNNSs [33], as shown in Figure 2b. The size, thickness, and crystallinity of the resulting BNNSs depend strongly on milling parameters such as duration, atmosphere, and milling additives. Moreover, the addition of suitable solvents during the ball milling process, i.e., solvent-assisted ball milling, significantly enhances exfoliation efficiency. Suitable solvents help prevent restacking, improve the dispersibility of BNNSs, and can even introduce functional groups onto their surfaces [34,35,36]. For example, urea-assisted ball milling yields few-layer h-BN nanosheets functionalized with amino groups at a high production yield of 80% and stable aqueous dispersions with a high concentration up to 30 mg/mL [34]. Ball milling in the presence of tannic acid (TA) produces modified BNNSs with large lateral size (~3.4 um) and excellent water solubility (40 mg/mL) [36].

Ball milling is simple, low-cost, and highly scalable, enabling gram-scale production. BNNSs produced via ball milling generally exhibit lateral sizes from several tens of nanometers to micrometers, with thicknesses spanning from a few atomic layers to several tens of layers. However, the intense mechanical forces inevitably introduce structural defects, edge damage, and partial amorphization, which may degrade the intrinsic thermal or mechanical performance of BNNSs.

#### 3.1.3. Liquid-Phase Exfoliation

Liquid-phase exfoliation involves dispersing bulk h-BN in an appropriate solvent and applying ultrasonic energy or shear forces to overcome the interlayer vdW interactions, thereby exfoliating them into few-layer or even monolayer nanosheets. Efficient exfoliation critically depends on matching the surface tension of the solvent with the surface energy of h-BN, which minimizes the interfacial energy penalty associated with the formation of new nanosheet–solvent interfaces. Common solvents such as N,N-dimethylformamide (DMF) [37], isopropyl alcohol (IPA) [38], and water [39] have been employed for BNNS exfoliation. Further, mixed-solvents such as ethanol/water mixture [40] and IPA/water mixture [41] have been shown to significantly enhance the exfoliation efficiency and production yield. Moreover, thermal assistance can further promote exfoliation by increasing kinetic energy of solvent molecules and providing additional energy to overcome interlayer interactions. Following exfoliation, centrifugation or filtration is usually employed to remove unexfoliated particles and to sort the BNNSs according to thickness and lateral size.

Despite these advances, the efficiency of conventional liquid-phase exfoliation remains intrinsically limited. In most processes using common solvents (e.g., DMF, NMP, and IPA), the exfoliation yield rarely exceeds 10% and the resulting suspension concentration of BNNSs is typically below 0.1–0.5 mg/mL [42], even after prolonged sonication and careful solvent optimization. These modest values primarily arise from the strong interlayer interactions in h-BN, which are reinforced by partial ionic B-N bonding and result in a pronounced tendency toward restacking, as well as from the chemical inertness of the basal plane, which hinders effective solvent–surface interactions. Moreover, exfoliation driven solely by mechanical energy is intrinsically governed by a pronounced trade-off between nanosheet quality and exfoliation yield. Under mild sonication or low-shear conditions, the applied energy is insufficient to overcome the exfoliation barrier for most crystallites, leaving a large fraction of the starting material unexfoliated. Conversely, aggressive exfoliation conditions inevitably introduce structural defects, reduce lateral dimensions and broaden the thickness distribution of the resulting BNNSs.

To overcome these limitations, chemically assisted exfoliation strategies have been developed, in which intercalation or mild functionalization is introduced prior to or during exfoliation. As illustrated in Figure 2c, intercalation-assisted exfoliation involves the insertion of cations, such as Li^+^, K^+^, or organic ammonium cations, into the vdW gaps of h-BN. This process increases the interlayer spacing, substantially weakens interlayer interactions, lowers the delamination energy barrier, and enables subsequent ultrasonic or mechanical shear forces to more effectively separate the layers into nanosheets [43,44,45,46,47]. The cation intercalation can be achieved under hydrothermal conditions at ~180 °C and a pressure of ∼2 × 10^6^ Pa, driven by the high chemical potential [44]. For example, the molten alkali-assisted exfoliation has been reported to yield BNNSs with an average yield up to 19%, although the suspension concentration of remains relatively low (~0.1 mg/mL) [47].

In addition, edge-selective functionalization, typically involving the introduction of hydroxyl or amino groups, can significantly enhance exfoliation efficiency by increasing the surface polarity of BNNSs, improving solvent compatibility, and suppressing restacking through electrostatic or steric stabilization. For example, exfoliation of h-BN using the highly reactive Lewis acidic chlorinating agent SOCl_2_ preferentially targets undercoordinated edge sites, forming B–Cl and B–OH species. This modification strengthens BNNS-solvent interactions and leads to an exfoliation yield up to 20% [48]. Similarly, exfoliation in monoethanolamine (MEA) aqueous solutions benefits from the presence of amino (–NH_2_) and hydroxyl (–OH) groups, which enable bonding with B-N bonds and Lewis acid–base interactions at edge sites. These interactions facilitate layer delamination and effectively suppress restacking, resulting in a high yield up to 42% and a suspension concentration as high as 1.5 mg/mL [42].

By combining intercalation with controlled functionalization, both exfoliation yield and suspension concentration can be dramatically enhanced, with ionic liquids emerging as particularly effective exfoliation media [49,50,51,52]. The ionic liquids consist entirely of ions and can intercalate into the vdW gaps of h-BN, thereby facilitating efficient layer separation. In addition, cations and anions in ionic liquids can physically and chemically adsorb onto BNNS surfaces and edges, forming stabilizing solvation shells around individual nanosheets, and introducing electrostatic repulsion that suppresses aggregation. Further, many ionic liquids inherently possess surface tensions in the range of 30–50 mN·m^−1^ [50,53], which closely match the surface energy of BNNSs. The synergistic effects of interlayer intercalation, surface adsorption-induced stabilization, and surface-tension matching enable ionic liquids to function as active exfoliation media capable of both high efficient exfoliation and high-concentration stabilization of BNNS dispersions [50,51]. For example, exfoliation in a mixture of [bmim][PF_6_] yields a highly stable BNNS suspension with a concentration up to 1.9 mg/mL at a yield up to ∼50% [50]. Even higher performance has been reported using a mixture of X_2_SiF_6_/NaOH, where a yield up to 78.5% (Li_2_SiF_6_/NaOH) and a suspension concentration up to 12.78 mg/mL ((NH_4_)_2_SiF_6_/NaOH) have been achieved [51].

Overall, liquid-phase exfoliation is a scalable and versatile approach capable of producing high-quality BNNSs with relatively large lateral sizes, offering a practical balance between efficiency and cost. Nevertheless, this method still suffers from several intrinsic limitations. In addition to the toxicity, environmental concerns, and removal difficulty associated with some commonly used organic solvents, conventional liquid-phase exfoliation typically requires prolonged ultrasonication to achieve effective exfoliation, and the exfoliation yield and suspension concentration of BNNSs remain relatively low, necessitating the use of large solvent volumes to obtain a sufficient amount of exfoliated material. These factors not only increase energy consumption and processing time but also hinder scalability and cost-effectiveness for large-scale production and downstream integration.

### 3.2. Bottom-Up Approaches

Top-down exfoliation is effective but often suffers from limited control over layer number, lateral size, and defect density. By contrast, bottom-up synthesis builds BNNSs directly from molecular precursors, allowing precise control over crystallinity, thickness, morphology, and uniformity. Representative bottom-up strategies include chemical vapor deposition (CVD) and metal–organic chemical vapor deposition (MOCVD).

#### 3.2.1. Chemical Vapor Deposition

**Figure 3:**
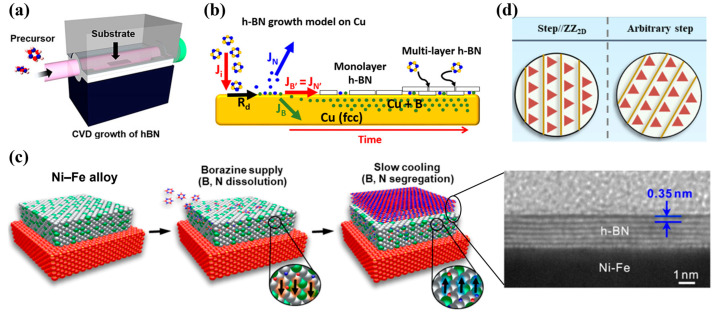
(**a**) Schematic diagram of CVD growth of h-BN [55]. (**b**) Schematic diagram of the mechanism governing h-BN growth on polycrystalline Cu substrate [61]. (**c**) Schematic illustration of the multilayer h-BN growth on a Ni-Fe alloy, along with cross-sectional TEM images of the resulting h-BN layers [64]. (**d**) Schematic diagrams showing h-BN growth on Cu(111) with steps parallel to the zigzag edge of h-BN (**left**) and with steps along arbitrary direction (**right**), indicating edge-dominated unidirectional alignment of h-BN domains [67].

CVD is one of the most powerful and widely used techniques for synthesizing high-quality 2D materials, including h-BN [54,55]. The CVD process involves thermal decomposition or reaction of gaseous precursors on a heated substrate (Figure 3a), where atoms or molecular fragments nucleate and grow into crystalline h-BN layers.

For the CVD growth of h-BN, the precursors are generally classified into single-source and dual-source B/N compounds according to whether boron and nitrogen are provided from the same or separate chemical species. Single-source precursors, such as borazine (B_3_N_3_H_6_) [56] and ammonia borane (NH_3_-BH_3_) [57], enables a stoichiometric release of boron and nitrogen species upon thermal decomposition. Dual-source precursors include a boron-containing compound, such as boron trichloride (BCl_3_) and diborane (B_2_H_6_), and a nitrogen source, such as ammonia (NH_3_) and nitrogen gas (N_2_) [58,59]. The reaction between these species at high temperature produces *sp*^2^ bonded h-BN nanosheets.

The substrate plays a decisive role in determining nucleation density, domain size, crystallographic orientation, growth mode, thickness and quality of the resulting h-BN films [7,24,25,54,55,60]. The influence of the substrate arises from its catalytic activity, surface energy, lattice structure, and solubility for boron and nitrogen, leading to different growth mechanisms such as catalytic growth, edge-limited growth, and vdW epitaxy.

Metal substrates, including Cu, Ni, Fe, Co, Pt, and their alloys, exhibit catalytic activity that promotes decomposition of boron- and nitrogen-containing precursors at relatively low temperatures. The growth mechanism depends strongly on the metal’s solubility for boron and nitrogen. Low-solubility substrates such as Cu favor surface-mediated growth and largely yield self-limiting monolayers (Figure 3b) [59,61]. High-solubility metals such as Ni or Fe enable a segregation–precipitation mechanism, where boron and nitrogen dissolve into the bulk during growth and later precipitate upon cooling, producing multilayer h-BN films [62]. Alloy substrates such as Cu-Ni or Ni-Fe enable tunable solubility and catalytic behavior for controlling over layer thickness and domain size (Figure 3c) [63,64]. Moreover, step-engineered or single-crystal metals (e.g., Cu(110), Cu(111), Pt(111)) further dramatically improve alignment, producing single-crystal h-BN film over centimeter-scale areas (Figure 3d) [65,66,67].

Dielectric substrates such as SiO_2_/Si, sapphire (Al_2_O_3_), SiC, and AlN function differently during h-BN CVD growth [68,69,70]. Because these substrates possess little to no catalytic activity for precursor decomposition, high-quality h-BN growth often requires elevated temperatures or the introduction of catalytic interlayers. On sapphire, particularly the (0001) surface, strong substrate–film interactions can enable well-aligned epitaxial growth through vdW epitaxy, where the surface lattice symmetry facilitates oriented BN nucleation and promotes the formation of wafer-scale single-crystalline films under optimized conditions [69]. In contrast, SiO_2_ is amorphous and chemically inert, leading to high nucleation densities and typically polycrystalline h-BN domains [68]. Despite this limitation, SiO_2_ remains technologically critical due to its broad integration in semiconductor platforms.

The choice of substrate fundamentally shapes BN nucleation behavior, growth kinetics, bonding configurations, and the resulting film morphology. A clear understanding of substrate-dependent growth mechanisms, such as catalytic growth on metals and vdW epitaxy on crystalline dielectrics, is therefore essential for advancing wafer-scale, high-quality h-BN films.

The CVD can produce wafer-scale uniform h-BN films with controlled thickness ranging from monolayers to tens of layers and excellent crystalline quality, making it suitable for electronic and photonic applications. However, the need for polymer-assisted or etching-based transfer remains a key limitation for CMOS integration.

#### 3.2.2. Metal–Organic Chemical Vapor Deposition

MOCVD offers a highly scalable platform for producing uniform h-BN films with exceptional step coverage, making it particularly compatible with semiconductor manufacturing [54,71,72,73,74]. High-volatility organometallic precursors, such as triethylboron (TEB), trimethylboron (TMB), boron trichloride (BCl_3_), or amine–borane complexes, combined with ammonia (NH_3_) or plasma nitrogen sources, can be delivered with precise flow control, enabling fine adjustment of growth chemistry and B/N composition. These precursors undergo thermal or plasma-assisted decomposition at the heated substrate surface, forming *sp*^2^ bonded h-BN layers.

A key advantage of MOCVD lies in its ability to achieve conformal h-BN growth on high-aspect-ratio structures, including trenches, vias, and sidewalls. Unlike conventional CVD, which is often mass-transport-limited and governed by island nucleation and lateral expansion, MOCVD typically proceeds in a reaction-limited regime. In this regime, slower and more uniform surface reactions allow precursor species to effectively reach and react on all facets of complex three-dimensional structures. However, achieving monolayer or few-layer h-BN remains challenging, and decomposition of metal–organic precursors may generate carbon-containing by-products, potentially introducing impurities or defects into the h-BN lattice.

**Table 1:** Comparison of methods for obtaining BNNSs.

Methods	Typical Lateral Size of BNNSs	Typical Thickness	Advantages	Disadvantages	Applications
Tape exfoliation	10–100 μm	mono- to few- layer	highest quality, simple and low-cost	not scalable, poor dimensional control	intrinsic properties, fundamental studies, proof-of-concept devices
Ball milling	50 nm–1 μm	few- to tens-of- layers	scalable, simple and low-cost	structural defects, poor dimensional control	composite fillers, heat-dissipation coatings, solid lubricants
Liquid-phase exfoliation	100 nm–5 μm	mono- to few- layer	scalable, high quality, versatile	time consuming, poor dimensional control	composite fillers, heat-dissipation coatings, ink-based printing
CVD	mm- to wafer-scale	mono- to few- layer	large-area film, desired thickness control	high cost, transfer bottleneck,	wafer-scale electronics, dielectric layers; heterostructures
MOCVD	wafer-scale continuous films	few-layer to um-scale	good uniformity control, industry-compatible	high cost, grain boundaries and defects	dielectric layers, passivation layers, heterostructures

## 4. Applications of BNNSs in Thermal Management

BNNSs combine exceptional thermal, electrical, chemical, and mechanical properties, making them highly promising for advanced thermal management. Their high in-plane thermal conductivity, ultralow dielectric constant, and intrinsic stability have led to extensive exploration as thermally conductive fillers in polymers and as key components in thermal interface materials (TIMs) [13,26,30,75,76,77,78,79,80].

**Figure 4:**
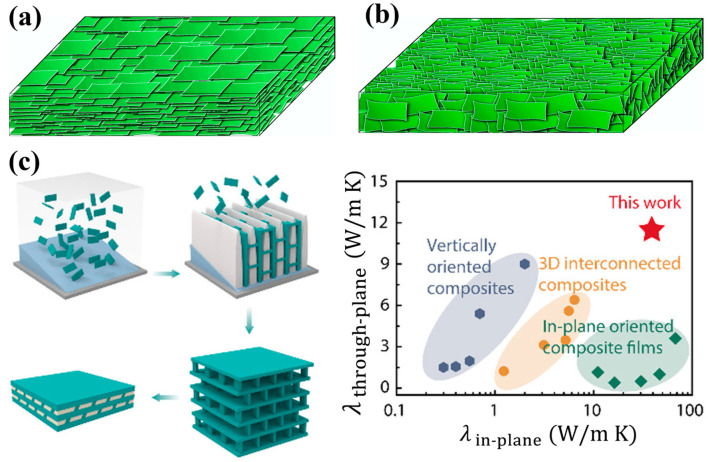
(**a**,**b**) Schematic diagrams of laminated (**a**) and vertically aligned (**b**) BNNSs films [82]. (**c**) Schematic diagrams of fabrication of biaxially oriented composite (**left**), and comparison of thermal conductivity with other reported BNNSs-based composite films (**right**) [94].

BNNSs can be assembled into freestanding films, and controlling nanosheet alignment is critical for maximizing thermal performance. In one common configuration, as shown in Figure 4a, BNNSs lie parallel to the substrate to form densely packed laminated structures, typically fabricated by vacuum filtration, blade coating, or spray coating. Such BNNS laminates exhibit high in-plane thermal conductivity up to 20 W/m K [81], while maintaining very low through-plane thermal conductivity of ~0.4 W/m K [82]. Such pronounced thermal anisotropy makes laminated BNNSs films particularly attractive for applications that require efficient lateral heat spreading in combination with robust electrical insulation [82]. In another configuration, as shown in Figure 4b, BNNSs can be vertically aligned such that their basal planes are oriented approximately perpendicular to the macroscopic surface. Vertically aligned architectures are typically achieved via directional freeze casting [83], electric-field-assisted alignment [84], or mechanical stretching [85], and they enable significantly enhanced through-plane thermal conductivity, making these structures highly suitable for heat dissipation across device interfaces.

Beyond their direct use as TIMs, BNNSs also serve as highly effective thermally conductive fillers for polymer composites. Most polymers inherently possess low thermal conductivity (<0.5 W/m K), severely constraining their deployment in high-power electronic systems [86]. Incorporating BNNSs into polymer matrices provides a practical approach for constructing continuous thermally conductive pathways without sacrificing mechanical flexibility, dielectric performance, or chemical stability [75,76,77,87]. Moreover, the large aspect ratio and two-dimensional morphology of BNNSs promote efficient heat-transfer networks at much lower filler loadings than required for spherical or rod-like fillers [88]. Numerous studies have shown that BNNSs/polymer composites can achieve thermal conductivities of 5–10 W/m K at moderate filler loadings of ~30–40 wt% [89,90,91,92]. When using BNNSs with large lateral dimensions of ~5.9 um, aramid nanofiber (ANFs)/BNNSs composites have demonstrated ultrahigh thermal conductivities exceeding 30 W/m K [93].

Alignment engineering plays an essential role in further optimizing thermal transport. Aligning the basal planes of BNNSs along the primary heat-flow direction minimizes phonon scattering and enhances the continuity of thermal pathways. Such alignment transforms the composite microstructure from an isotropic system into a strongly anisotropic architecture, either layered or vertically oriented, greatly improving directional thermal conductivity. For example, through-plane thermal conductivity up to 9 W/m K has been achieved in vertically aligned BNNSs/epoxy (EP) composites with a 44 vol% filler loading [95]. The in-plane aligned BNNSs embedded in polyvinylidene fluoride (PVDF) yield a polymer composite with high in-plane thermal conductivity of 16.3 W/m·K at 33 wt % loading [96]. More complex dual-direction alignment strategies (Figure 4c) have enabled composites exhibiting both ultrahigh in-plane (∼39.0 W/m K) and through-plane (∼11.5 W/m K) thermal conductivity at 80 vol% loading [94].

Although the thermal conductivity of BNNS/polymer composites can be significantly enhanced by incorporating BNNS fillers, the achieved values are often far below the theoretical limit predicted from the intrinsic thermal conductivity of BNNSs. This discrepancy is primarily attributed to the large interfacial thermal resistance within the composite. Phonon scattering at filler–matrix interfaces, together with weak interfacial coupling, severely impedes heat transport across the composite, thereby limiting the effective utilization of the intrinsic thermal conductivity of BNNSs [75,78,79,97,98].

Interfacial engineering between BNNSs and the polymer matrix has proven to be an effective strategy for reducing thermal resistance and enhancing phonon transport [89,99]. Surface functionalization of BNNSs can strengthen interfacial interactions by introducing covalent bonds, hydrogen bonding, or strong Lewis acid–base interactions with polymer chains, thereby improving phonon transmission across the interface. Notably, controlled surface functionalization preserves the intrinsic thermal conductivity of BNNSs. For example, the interfacial thermal resistance between APTES-functionalized BNNSs (APTES-BNNSs) and an epoxy matrix (1.88 × 10^−9^ m^2^·K·W^−1^) is an order of magnitude lower than that between non-functionalized BNNSs and epoxy (3.32 × 10^−8^ m^2^·K·W^−1^). As a result, the thermal conductivity of the APTES-BNNSs/epoxy composite reaches 5.86 W/m·K, almost twice that of the BNNSs/epoxy composite (3.03 W/m·K) [89]. Similarly, amino-acid-functionalized BNNSs (Trp-BNNSs) exhibit a substantially reduced interfacial thermal resistance with the epoxy matrix (2.09 × 10^−8^ m^2^·K·W^−1^), which is approximately one-third of that of the unmodified BNNSs/epoxy interface (6.7 × 10^−8^ m^2^·K·W^−1^). Correspondingly, the thermal conductivity of the Trp-BNNSs/epoxy composite (2.1 W/m·K) is nearly three times higher than that of the BNNSs/epoxy composite (0.89 W/m K) [99].

**Figure 5:**
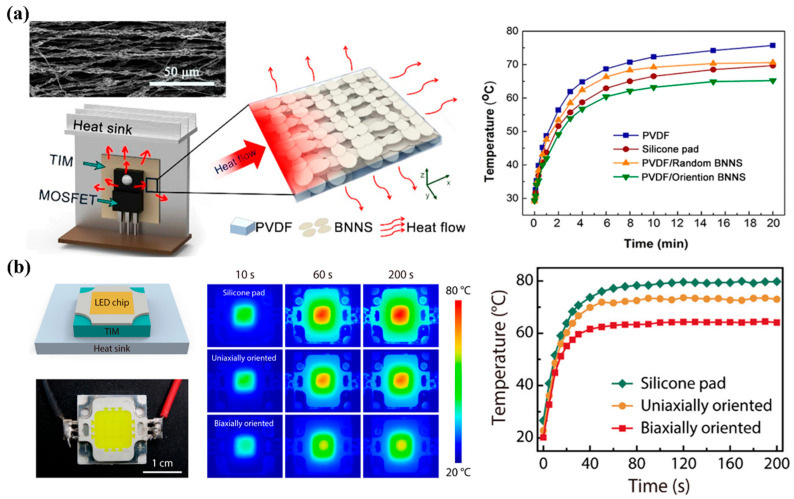
(**a**) Schematic structure of PVDF/BNNSs nanocomposite TIM films (**left**), and the corresponding MOSFET temperature evolution with different TIMs (**right**) [96]. (**b**) Schematic and optical images of the biaxially oriented BNNSs TIM placed between the LED chip and heat sink (**left**), infrared images of LED chips using three TIMs including silicone pad, uniaxially aligned composite, and biaxially aligned composite (**middle**), and the corresponding temperature evolution of the LED chips (**right**) [94].

Leveraging these enhanced thermal properties, BNNSs-based polymer composites have emerged as strong candidates for next-generation TIMs in high-power electronics and advanced semiconductor packaging. As shown in Figure 5a, using BNNS/PVDF composites as TIMs between a MOSFET and a heat sink reduces the device surface temperature by 11 °C and 5 °C relative to pure PVDF films and commercial silicone pads, respectively [96]. Likewise, a biaxially oriented BNNSs/polyurethane (PU) composite used as TIMs for LED packaging lowers the LED operating temperature by up to 15 °C compared with commercial silicone pads (Figure 5b) [94]. These demonstrations highlight the superior heat-dissipation capabilities of BNNSs-based composites, underscoring their significant potential in next-generation thermal management applications.

## 5. Conclusions and Perspectives

BNNSs represent a distinctive class of two-dimensional materials that combine ultrahigh intrinsic thermal conductivity, excellent electrical insulation, outstanding chemical stability, and remarkable mechanical robustness. These attributes position BNNSs as one of the most promising candidates for next-generation thermal management. This review provides an overview of the crystallographic structure, and key electrical, thermal, chemical and mechanical properties of h-BN/BNNSs. We further compare the major synthesis strategies, including top-down exfoliation techniques (tape exfoliation, ball milling, liquid-phase exfoliation) and bottom-up synthesis approaches (CVD and MOCVD), each offering unique advantages in controllability, sheet size, structural quality, and scalability. Finally, we examine the rapidly expanding application landscape of BNNSs, with particular emphasis on their roles as high-performance thermally conductive fillers and thermal interface materials. Collectively, these developments clearly demonstrate strong application potentials of BNNSs for electronic packaging, power electronics, and next-generation semiconductor systems.

Despite notable progress, several challenges remain before BNNSs can be widely adopted in industrial thermal management technologies.

1. Scalable synthesis of large-area, high quality BNNSs.

Achieving large-scale production of BNNSs with ultrathin thickness, large lateral size, and high structural integrity remains a central challenge for their practical deployment in advanced thermal management and electronic systems. Top-down exfoliation methods can produce BNNSs with desirable thickness, but they are hindered by low exfoliation efficiency and heavy reliance on organic solvents. Bottom-up synthesis techniques enable wafer-scale films with good uniformity and structural integrity, but they face critical bottlenecks including high energy consumption, stringent growth conditions, and limited throughput. Overall, overcoming these challenges is essential for transitioning BNNSs from laboratory-scale demonstrations to industrial-level manufacturing and widespread technological applications.

2. Advanced alignment engineering in polymer matrices.

Achieving programmable, multi-directional alignment of BNNSs within polymer matrices is essential for next-generation thermal management but remains technically challenging. The main obstacles include process compatibility with scalable manufacturing (roll-to-roll, extrusion, or printing) while avoiding filler damage or aggregation, and the trade-off between achieving high degree of alignment and maintaining large lateral size and dispersion of BNNSs. Integrating alignment techniques with in-line metrology and scalable process windows will be critical to the manufacturable BNNS/polymer TIMs.

3. Interface engineering for BNNSs and polymer composites.

Interfacial thermal resistance (ITR) is the primary bottleneck limiting heat-transfer performance in BNNSs/polymer composites. Despite the high intrinsic thermal conductivity of BNNSs, phonon scattering at mismatched BNNSs/polymer interfaces greatly reduces effective composite thermal conductivity. Current understanding is further constrained by limited experimental tools for probing nanoscale interfacial heat transport. Chemical functionalization and surface modification of BNNSs can significantly strengthen interfacial bonding and markedly reduce interfacial barriers, while tailored functional groups enhance compatibility with specific polymer matrices. Future progress demands improved theoretical models and advanced nanoscale thermal-characterization techniques to elucidate interfacial phonon processes and guide rational design of high-performance thermal interface materials.

## Data Availability

The original contributions presented in this study are included in the article. Further inquiries can be directed to the corresponding author.
